# Large red cell-derived membrane particles are major contributors to hypercoagulability in sickle cell disease

**DOI:** 10.1038/s41598-021-90477-z

**Published:** 2021-05-26

**Authors:** Rachel A. Smith, Tosti J. Mankelow, Despoina Drizou, Thomas Bullock, Tom Latham, Sara Trompeter, Allison Blair, David J. Anstee

**Affiliations:** 1 Bristol Institute for Transfusion Sciences, National Health Service Blood and Transplant, Northway, Filton, Bristol, BS34 7QH UK; 2 NIHR Blood and Transplant Research Unit, University of Bristol, Bristol, UK; 3 School of Biochemistry, University of Bristol, Bristol, UK; 4 School of Cellular and Molecular Medicine, University of Bristol, Bristol, UK; 5 Joint Red Cell Unit, Haematology Department, University College London National Health Service Foundation Trust, London, UK

**Keywords:** Sickle cell disease, Mechanisms of disease

## Abstract

Sickle cell disease (SCD) is one of the most common inherited single gene disorders. Polymerisation of sickle hemoglobin results in erythrocytes that are inflexible and adherent, leading to coagulation, vascular and cellular activation and resultant blood vessel blockage. Previous studies have observed elevated numbers of red cell-derived particles (RCDP), also denoted extracellular vesicles, in SCD plasma. Here, imaging flow cytometry was used to quantify all RCDP in SCD plasma. A more heterogenous population of RCDP was observed than previously reported. Significantly, large right side-out red cell macrovesicles (MaV), 7 µm in diameter, were identified. Most RCDP were right side-out but a minor population of inside-out vesicles was also present. Electron micrographs confirmed the heterogenous nature of the RCDP detected. All MaV are decorated with prothrombotic phosphatidylserine (PS) and their removal from plasma lengthened clotting times by more than three-fold. Removal of all right side-out RCDP from SCD patient plasma samples resulted in a seven-fold increase in clotting time. These results indicate that MaV comprise a large area of prothrombotic membrane and are thus major contributors to hypercoagulation in SCD. Consequently, controlled removal of MaV and PS exposed RCDP from plasma could provide a novel therapy for managing this disease.

## Introduction

Sickle cell disease (SCD) is characterised by hemolytic anaemia, hypercoagulability and inflammation. This complex pathology results from the inheritance of homozygosity for a single base change in the beta globin gene (Glu to Val at codon 6) which creates hemoglobin capable of polymerisation at low oxygen concentrations^1^. Repeated reversible polymerisation events result in hemolysis, releasing free hemoglobin and extracellular vesicles (EV) into the circulation^2^. Hypercoagulation plays a major role in the pathology of SCD^3–7^. Erythrocyte-derived EV, like other EVs, are prothrombotic due to surface expression of phosphatidylserine (PS)^8–13^. In addition, entrapped hemoglobin scavenges nitric oxide preventing it from performing its protective functions of vasodilation, inflammatory regulation and inhibiting platelet activation^14^. Previous studies, using conventional flow cytometry and antibodies to the extracellular domain of glycophorin A (GPA), show that SCD patients have increased levels of circulating red cell-derived EV compared to healthy controls^9,10,15,16^. These studies assume that there is just a single homogenous population of EV formed by erythrocyte membrane budding during sickling that have the same membrane orientation as the erythrocyte plasma membrane (right side-out EV)^2^. However, the presence of inside-out autophagic vesicles (AV), released during disease altered reticulocyte maturation, suggests the red cell-derived EV population must be more heterogenous^17^.

In this study we use imaging flow cytometry in association with antibodies to both extracellular and intracellular GPA epitopes to identify and quantify all red cell-derived EV in SCD plasma. In addition to EV and AV, we describe the presence of two previously unrecognised populations of red cell-derived particles, large PS-exposed red cell macrovesicles (MaV) and unsealed erythrocyte membrane fragments in SCD patients during steady state and crisis. As the membrane fragments and MaV are not true EV, the term red cell-derived particles (RCDP) is used to describe all membranous particles derived from erythrocytes that are present in plasma. Immunoaffinity removal of RCDP subpopulations was used to determine the relative contribution of each to hypercoagulability. These findings indicate that PS-exposed MaV are major contributors to hypercoagulation and that selective removal of RCDP from plasma could offer a novel transfusion independent therapeutic treatment for this debilitating disease.

## Results

Previous studies utilised antibodies that recognise an extracellular epitope on GPA to distinguish RCDP from those derived from other cells^9,10,15,16^. However, these antibodies would not detect AV that have an inside-out orientation (an opposite membrane orientation to that of the erythrocyte plasma membrane) as they present the cytoplasmic domain of GPA on their surface. We dual stained SCD plasma with BRIC256^18^, which recognises an extracellular epitope on GPA, and BRIC163^19^, which recognises an intracellular GPA epitope, in order to distinguish right side-out vesicles (BRIC256^+ve^) and inside-out AVs (BRIC163^+ve^)^17^. Imaging flow cytometry permitted visualisation of all RCDP (see Supplementary Fig. 1 for gating strategy) including detection of microparticles too small to be detected on a conventional flow cytometer.

**Figure 1:**
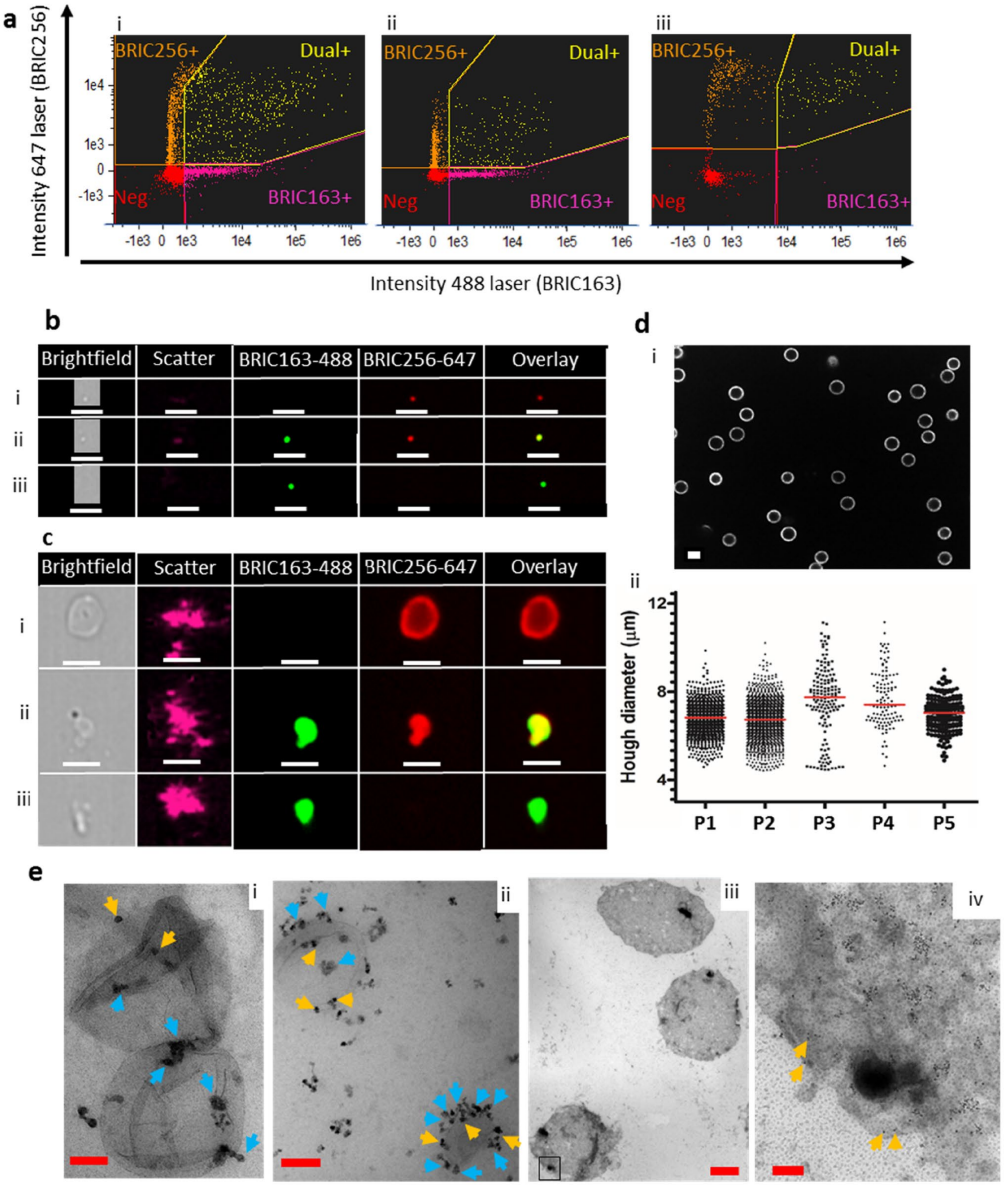
Visualisation and quantification of RCDP from SCD PFP. SCD plasma was processed and stained with BRIC256-Alexa Fluor 647 and BRIC163-Alexa Fluor 488, as described in the materials and methods. (**a**) Analysis of all RCDP detected (i) revealed the presence of BRIC256^+ve^/163^−ve^ stained particles (right-side out), BRIC256^-ve^/163^+ve^ stained particles (inside-out) and BRIC256^+ve^/163^+ve^ dual stained particles (membrane fragments). Further analysis split the RCDP into (ii) low scatter (small size) and (iii) high scatter (large size). Images of (**b**) low scatter and (**c**) high scatter of (i) BRIC256^+ve^/163^−ve^, (ii) dual BRIC256^+ve^/163^+ve^ and (iii) BRIC256^−ve^/163^+ve^ RCDPs. White scale bars represent 7 µm. (**d**) Spinning-disk confocal analysis of BRIC256^+ve^/BRIC163^−ve^ sorted steady state SCD samples. (i) Spinning-disk confocal image. Scale bar 5 µm. (ii) Scatter plot of size of each individual round object detected in patient samples. Line represents median values. (**e**) Transmission electron microscopy was performed on enriched populations of right-side or inside-out RCDP, (i) Isolated right-side RCDP were stained using immunogold labelled extracellular AE-1 (BRIC200). Red scale bar indicates 100 nm. (ii) Inside out RCDP intracellular AE-1 (BRIC132) positive were stained with gold intracellular AE-1 (BRAC66) Red scale bar indicates 100 nm. (iii) MaV stained using immunogold labelled extracelluar GPA. Boxed area corresponds to area shown in (iv). Red scale bars indicate 2 μm (iii) or 200 nm (iv). Yellow arrows indicate immunogold particles and blue arrows indicate magnetic beads.

Our data show that platelet free plasma (PFP) from SCD patients contains a more heterogenous population of RCDP than previously described^9,10,15,16^, (Fig. 1). The most abundant population detected corresponded to BRIC256^+ve^/163^−ve^ particles. As expected^17^, BRIC256^−ve^/163^+ve^ AVs were also present along with dual BRIC256^+ve^/163^+ve^ particles (Fig. 1ai) which are likely to be unsealed membrane fragments from lysed red cells. Data were subdivided into events with low scatter (small particles, Fig. 1aii,b) and high scatter (larger particles, Fig. 1aiii,c). The low scatter particles were predominantly EV (Fig. 1b)^20^. The BRIC256^+ve^ low scatter events are likely EV released by cellular blebbing during sickling^2^. A large proportion of the high scatter BRIC256^+ve^/163^−ve^ events correspond to large (~ 7 µm), macrovesicles (MaV) (Fig. 1ci). Pictorial imaging flow cytometry data show MaV as ~ 7 µm, however, the use of size beads show that they have lower scatter intensity than 0.5 µm size beads (Supplementary Fig. 2). To clarify this disparity, we used spinning-disk confocal analysis to confirm the size of MaV. Our data show that MaV have a median diameter of 7 μm (range 6.8–7.8 μm) (Fig. 1d and Supplementary Table 2). Spinning-disk confocal imaging is not sensitive enough to detect the low scatter RCDP population or the smaller sized particles of the high scatter population, therefore, it only measures the size of the MaV population. All intact cells were removed from PFP by centrifugation (as described in materials and methods) and the membrane definition of MaV, as viewed by imaging flow cytometry, does not correspond to that of erythrocytes (Supplementary Fig. 3), so MaV are clearly not cellular remnants retained in the PFP. MaV are found in PFP from healthy individuals but in much lower numbers than SCD. The morphology of both the right-side and inside-out vesicle populations was examined by transmission electron microscopy (TEM). In both types of vesicles, round structures were observed with an external lipid bilayer (Fig. 1e). Magnetic beads (~ 50 nm) appeared as roundish, grey structures (blue arrows) and gold particles (~ 10 nm) as black dots (yellow arrows). The TEM data show that the right side-out and inside-out RCDP are structures with an intact plasma membrane. We believe this is the first study to show the presence of MaV, AV’s and unsealed membrane fragments circulating in SCD plasma.

**Figure 2:**
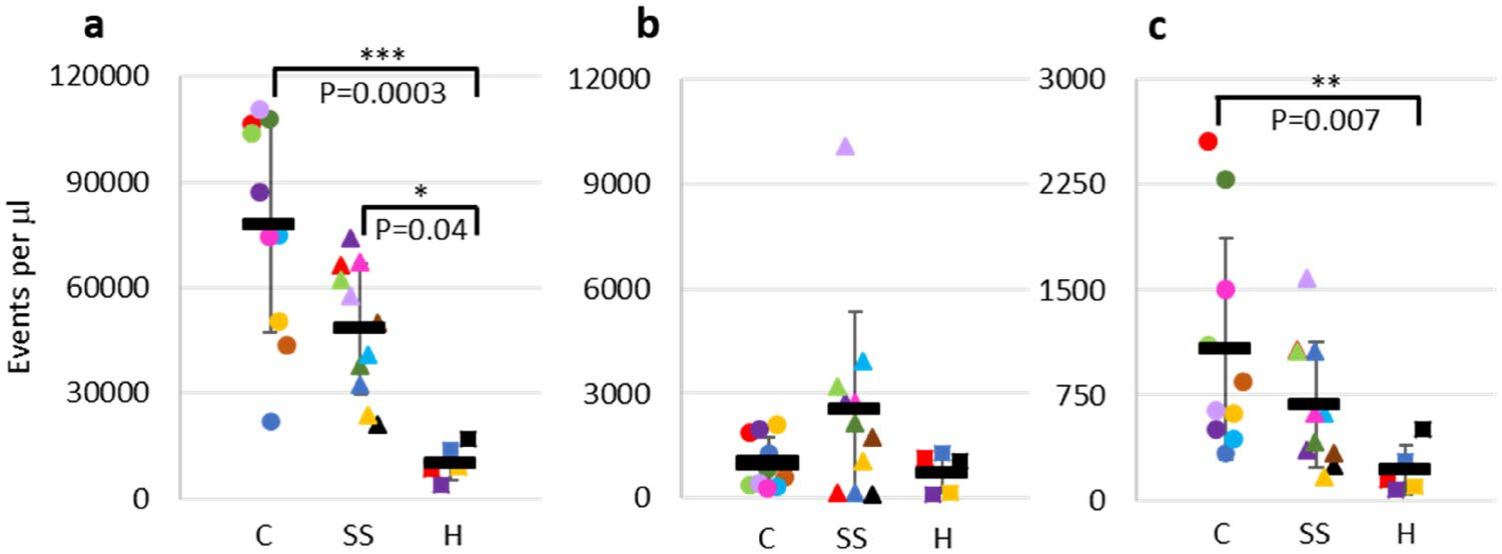
Analysis of RCDP in PFP from SCD patients in steady state and in crisis by imaging flow cytometry. PFP from SCD patients in steady state and in crisis and health individuals was stained with BRIC256-Alexa Fluor 647 and BRIC163-Alexa Fluor 488, as described in the materials and methods. Numbers of (**a**) BRIC256^+ve^/163^−ve^, (**b**) BRIC256^−ve^/163^+ve^ and (**c**) dual BRIC256^+ve^/163^+ve^ events found in SCD plasma from patients in crisis (circle, n = 10), steady state (triangle, n = 11) and from healthy individuals (square, n = 5). Each data point represents one sample with the black bars showing the mean number of events in each cohort along with standard deviation. Points with the same shape and colour are identical samples. Statistically significant differences are shown.

We analysed PFP from SCD patients in steady state and in crisis in comparison with PFP from healthy individuals (Fig. 2). The imaging flow cytometry dot plots from SCD PFP and healthy PFP show the differences in RCDP present (Supplementary Fig. 4). Steady state SCD patients have significantly elevated levels of all RCDP (low and high scatter) with fivefold more BRIC256^+ve^/163^−ve^ (right side-out) RCDP (*P* = 0.04) and approximately threefold more BRIC256^−ve^/163^+ve^ (inside-out) and dual BRIC256^+ve^/163^+ve^ (unsealed membrane fragments) RCDP than healthy controls. Patients in crisis had significantly higher levels of BRIC256^+ve^/163^−ve^ (*P* = 0.0003) (right side-out RCDP) and dual BRIC256^+ve^/163^+ve^ (membrane fragments) particles than healthy controls (*P* < 0.007). In this respect our imaging cytometry data mirrors previous studies, using conventional flow cytometry^11,21^, and validates imaging cytometry technology for such analyses.

**Figure 3:**
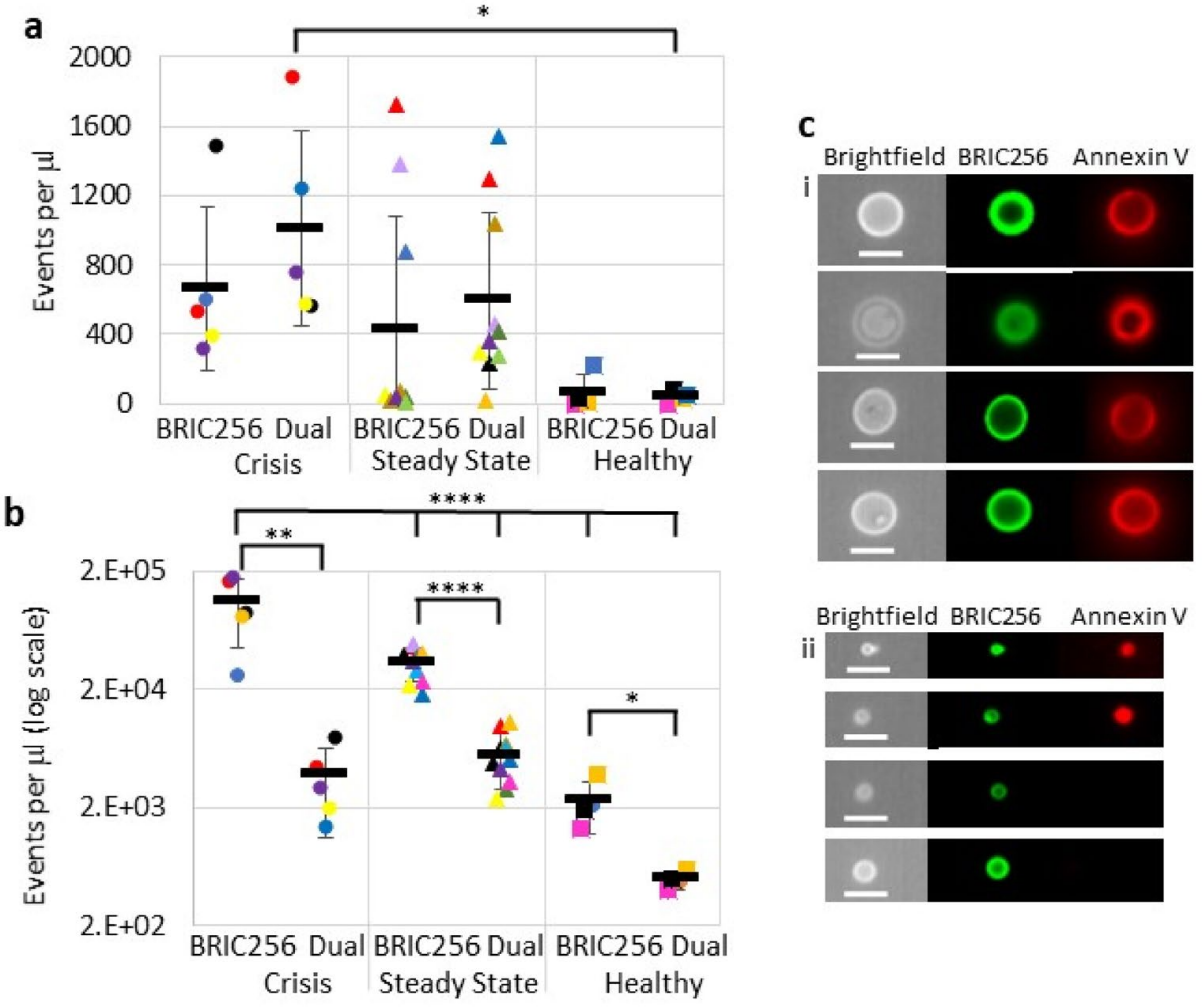
Phosphatidylserine decoration of RCDP. SCD plasma stained with BRIC256-Alexa Fluor 488 and Annexin V-Alexa Fluor 647, as described in the materials and methods. Shown is the analysed data for (**a**) high scatter events and (**b**) low scatter events. Numbers of BRIC256^+ve^ only and dual BRIC256^+ve^/Annexin V^+ve^ events found in SCD plasma from patients in crisis (circles, n = 5), steady state (triangles, n = 10) and from healthy individuals (squares, n = 4). Each symbol represents an individual patient sample. Bars represent mean number of events in each cohort ± standard deviation. Statistically significant differences are shown. (**a**) There is a significant difference between the number of dual BRIC256^+ve^/Annexin V^+ve^ RCDP in SCD patients plasma in crisis and healthy controls (P = 0.04). (**b**) There are significant differences between solely BRIC256^+ve^ and dual BRIC256^+ve^/Annexin V^+ve^ RCDP in plasma from each individual sample, regardless of source (crisis *P* = 0.0001, steady state *P* < 0.0001 and healthy *P* = 0.4). There were significantly more solely BRIC256^+ve^ events in patients in crisis compared to those in steady state and healthy controls (*P* < 0.0001). (**c**) Images of high scatter events showing (i) the purely dual BRIC256^+ve^/Annexin V^+ve^ MaV subpopulation and (ii) the mixed solely BRIC256^+ve^ and dual BRIC256^+ve^/Annexin V^+ve^ smaller RCDP. Scale bars 7 µm.

**Figure 4:**
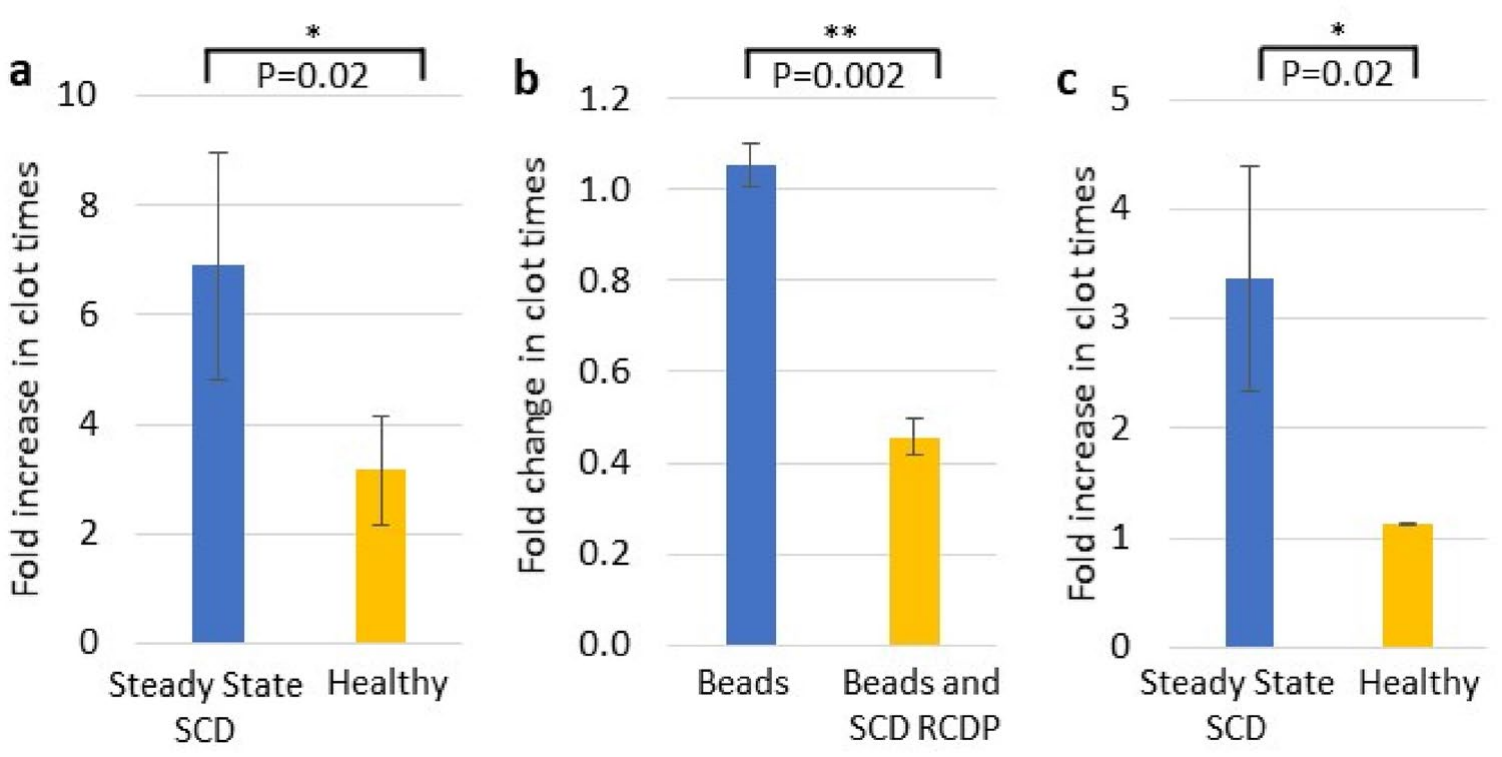
Phosphatidylserine decorated RCDP are major contributors to hypercoagulability in SCD. RCDP were selectively removed from SCD PFP using an anti-glycophorin A antibody attached to magnetic microbeads. After RCDP removal the clotting times were assayed (**a**) and found to increase (steady state SCD blue, healthy individual yellow). Average steady state SCD clotting times was 129.9 s and average healthy clotting times was 153.7 s. The amount of RCDP removal was; steady state SCD, 94.1% of all RCDP present at start and healthy 88.8% of all RCDP present at start. (**b**) When SCD RCDP are added to healthy PFP the clotting times are reduced (mean SCD RCDP addition to healthy PFP 3788 events per µl as assayed by imaging flow cytometry) (blue—healthy PFP with just microbeads and yellow—healthy PFP with SCD RCDP attached to microbeads). (**a**) and (**b**) n = 6 SCD samples and n = 3 healthy samples. (**c**) Healthy and steady state SCD PFP (n = 4 SCD samples and n = 2 healthy samples) was passed through a 1.2 µm filter and samples analysed by imaging flow cytometry to determine changes in RCDP (mean removal rates; steady state SCD, 1.9% of all low scatter RCDP present at start, 3.4% of all high scatter (not including MaV) RCDP present at start and 95.8% of all MaV present at start and healthy, 17.5% of all low scatter RCDP present at start, 0.83% of all high scatter (not including MaV) RCDP present at start and 68% of all MaV present at start) and clotting times found to increase after filtration (steady state SCD blue, healthy yellow).

Membrane exposure of PS is a major causative factor of hypercoagulation in SCD^22^. To establish whether the different populations of RCDP observed were PS^+ve^, PFP from SCD patients, in steady state and crisis, along with healthy controls were dual stained with BRIC256 and Annexin V (Fig. 3a–c). In order to demonstrate specificity of staining, Annexin V was added in the presence and absence of 2 mM EDTA (data not shown). The majority of the BRIC256^+ve^ high scatter events and all the MaV population are PS^+ve^ (Fig. 3a,c). Similar PS decorated large membrane structures to the MaV reported here have previously been observed by electron microscopy in low numbers in healthy plasma by Arraud et al.^23^ However, here we show their numbers are significantly elevated in the plasma of SCD patients in crisis compared to healthy subjects (*P* = 0.04). Low scatter BRIC256^+ve^ particles were more numerous, particularly in SCD patients in crisis (*P* < 0.0001, Fig. 3b), however, significantly fewer particles were also PS^+ve^, regardless of the plasma source (*P* ≤ 0.04). The failure of the low scatter small particles to bind Annexin V may be due to the high curvature of these sub-cellular particles rather than an absence of PS^24^. We investigated whether PS^+ve^ RCDP influenced PFP clotting times. Removing right side-out RCDP from SCD PFP (94.1% removal, Supplementary Fig. 5) significantly increased clotting times sevenfold (*P* = 0.02, Fig. 4a), while removing them from healthy PFP (88.8% removal, Supplementary Fig. 5) increased clotting times by only threefold (*P* = 0.02 compared to the increase observed in SCD plasma, Fig. 4a). Moreover, placing magnetic beads coated with right side-out RCDP from SCD PFP into PFP from healthy individuals decreased clotting times by half (*P* = 0.02), whereas addition of the magnetic beads alone had no effect (*P* = 0.002 compared to the decrease observed in SCD plasma, Fig. 4b). As all MaV express PS it was likely this sub-population of RCDP was highly prothrombotic, therefore, we assayed clotting times after filtration through a 1.2 µm filter to remove the MaV population but retain the other RCDP populations (Fig. 4c and Supplementary Fig. 6). There was variation in the proportions of the different RCDP removed by filtration between SCD and healthy controls, this is likely down to the much smaller number of RCDP found in the healthy controls to begin with (Supplementary Fig. 6C). Removal of the MaV population increased clotting times by over threefold (Fig. 4c, P = 0.03), approximately half the increase observed with removal of the entire RCDP population (Fig. 4a). In comparison, no effect was seen on the clotting time of healthy samples that had been passed through a 1.2 µm filter (*P* = 0.02 compared to that observed in SCD samples, Fig. 4c).

## Discussion

The primary defect in SCD involves the erythroid lineage and results in two major abnormalities, enhanced red cell destruction (hemolysis) with corresponding reticulocytosis. Previously the production of RCDP was thought a consequence of repeated sickling episodes, resulting from membrane blebbing producing right side-out EVs, which express surface exposed PS^10,11,15,16,21^. However, the production of inside-out AV during reticulocyte maturation indicated that potentially other processes are involved^17^. The presence of RCDP in the plasma of healthy individuals and in donated erythrocytes stored for transfusion shows that erythrocyte vesiculation is not unique to SCD. There is a possibility that a small number of RCDP observed in the plasma of the cohort of SCD patients studied here could have been generated from healthy erythrocytes they have received through transfusion. Identification and characterisation of RCDP in the plasma of SCD patients has generally employed conventional flow cytometry, however, in this study we used the more sensitive imaging flow cytometry. The results show that RCDP are present in a wide range of sizes from MaV and fragments of red cell membrane to small EVs. AVs are also present but appear to be far less abundant than the other subsets of RCDP (Fig. 2b). These data demonstrate that different types of RCDP, rather than a single homogenous population, circulate in plasma. As in previous studies, which used conventional flow cytometry, we show that there are significant differences in the numbers of RCDP present in the plasma of healthy individuals and SCD patients both in steady state and crisis^11,21^. SCD patients in crisis have significantly elevated numbers of BRIC256^+ve^/BRIC163^−ve^ right side-out RCDP and membrane fragments (dual BRIC256^+ve^/BRIC163^+ve^) in plasma, both likely formed by the sickling process and subsequent erythrocyte destruction that occurs in blood vessel occlusions during crisis (Figs. 1, 2).

A surprising finding of this study is the discovery of circulating MaV. Multiple centrifugations of the plasma samples (see “Methods”) ensured that the cellular components of blood were removed. As MaV were retained in the supernatant and they have a different membrane definition and different scatter properties when observed by the ImageStream to erythrocytes (Supplementary Fig. 3), then MaV are clearly not erythrocytes, despite expression of GPA on their surface. PS decorated, 6–8 µm, erythroid structures have previously been observed by electron microscopy in low numbers in healthy plasma by Arraud et al.^23^ and it is likely that these are analogous structures to the MaV reported here. Arraud et al. compared these large PS decorated structures to erythrocyte ghosts produced by osmotic lysis. However, we show that MaV are BRIC256^+ve^/BRIC163^−ve^ and thus must be intact structures with a right-side out membrane orientation. If they were lysed, they would stain with both BRIC256, with an extracellular epitope and BRIC163 which has an intracellular epitope. In addition, we show that MaV are present in much greater numbers in the plasma of SCD patients than the plasma of healthy individuals (Fig. 3a) and thus either their formation must be facilitated or their removal hindered by the SCD phenotype. MaV are likely to originate from erythrocytes that have been transformed in an unknown process, probably exacerbated by repeated sickling, into large membrane particles that have lost membrane phospholipid asymmetry. Initially we used size beads to confirm the visualised size of the MaV by imaging flow cytometry. However, as measured by scatter intensity, MaV appear as much smaller particles (between 0.2 and 0.5 µm, Supplementary Fig. 2). The pictorial sizing within the imaging flow cytometry data (Fig. 1c and Supplementary Fig. 3) alongside the spinning disc confocal data (Fig. 1d and Supplementary Table 1) and electron microscopy (Fig. 1e) clearly show MaV are much larger (~ 7 µm) than their scatter intensity indicates. We hypothesize the low scatter observed for MaV is either a result of hemoglobin loss and thus a decrease in intracellular complexity or alternatively because they have a dysfunctional and/or damaged cytoskeleton. Indeed, although similar to erythrocyte ghosts, MaV differ in respect to PS decoration. In ghosts, with an intact cytoskeleton, PS locates to the intracellular side of the membrane^25^, likely due to interactions with both alpha and beta spectrin^26^. However, as we observed with MaV (Fig. 3), in vesicles without a functional cytoskeleton PS asymmetry is lost. It is likely MaV have been previously reported as much smaller objects, detected on standard flow cytometers, in previous studies^9,10,15,16^. Membrane exposure of PS has long been known to be a major causative factor of the hypercoagulative state in SCD^22^. Red cell EVs produced in storage have been shown to directly activate coagulation pathways^12^, and evidence in mice suggests that coagulation is involved in vaso-occlusive crisis^13^. MaV are the least abundant population of RCDP in SCD plasma, however, due to their considerable diameters they present a large surface area of PS decorated membrane and so it is unsurprising that they have a pro-coagulative effect. These data suggest that PS decorated RCDP in plasma are prothrombotic and that in SCD, where there are large numbers of RCDP, particularly MaV, it equates to a substantial surface area of circulating prothrombotic membrane which significantly contributes to the hypercoagulative state (Fig. 3).

PS exposed membranes have been demonstrated to act as surfaces for complement deposition^27^, and thus activation of the immune system. As a result, we would hypothesize that these large PS exposed particles (MaV) as well as being pro-thrombotic, would also be pro-inflammatory and thereby contribute greatly to the pathology of SCD. Intravascular hemolysis is linked to inflammation and organ injury. Heme and heme-loaded vesicles induce alternative and terminal complement pathway activation resulting in complement deposition and vaso-occlusive events in the kidney^28–30^. Previous publications have associated RCDP with hypercoagulability^31^, and linked elevated levels of EVs to crisis^10^. We isolated RCDP from the plasma of SCD patients by immunoaffinity absorption and demonstrated a dramatic increase in clotting times. RCDP from SCD plasma also provoked a considerable shortening of clotting time when added to normal healthy plasma. Therefore, we have demonstrated a direct link between RCDP and hypercoagulation in SCD patients. We conclude removal of RCDP from the plasma of SCD patients has the potential to reduce the frequency and severity of vaso-occlusive episodes and provide a much-needed additional therapeutic intervention, improving the quality of life of patients with SCD. Such a procedure would be of particular benefit to patients for whom transfusion is not possible either because of the presence of multiple alloantibodies or previous hyperhemolysis or because of the lack of availability of a safe blood supply, the latter being the case for many patients with this condition worldwide.

## Methods

### Patients

SCD Patients presenting to University College London Hospitals (UCLH) in a ‘steady state’ or ‘in crisis’ were eligible for inclusion in the study. Steady state was defined as “attending the clinic for a routine red cell exchange transfusion and not having had a transfusion in the previous 4 weeks”. In crisis was defined as “presenting to the Emergency Department in pain”. Blood samples were drawn immediately prior to any transfusion procedure and anonymised. All patients gave informed written consent and the study was approved by NRES Committee London (Harrow). Experimentation was performed in accordance with the Declaration of Helsinki and within relevant institutional and ethic committee guidelines. Plasma used for electron microscopy and spinning disk confocal analyses came from surplus blood from anonymised samples taken from patients in steady state for clinical haematological analysis from SCD patients presenting at University Hospitals Bristol and Weston NHS Foundation Trust (UHBWT).

### Samples

Blood samples from UCLH were drawn into vacutainers containing sodium citrate. Within 1 h, plasma and cells were separated after centrifugation (2000*g* for 15 min at room temperature with no brake) and the plasma stored at 4 °C for under 48 h. After transportation the plasma was further centrifuged twice (2500*g* for 20 min at room temperature) and the pellet discarded after each step. Approximately 100 µl supernatant was left in the tube after each centrifugation to avoid disturbing the pellet. The PFP was aliquoted and stored at − 80 °C until analysis when it was thawed at 37 °C and used immediately. Samples from UHBWT were processed in an identical manner once clinical haematological analysis had been completed. Control samples were drawn from healthy volunteers and processed identically. In total 10 crisis and 17 steady state blood samples were obtained from 10 SCD patients in crisis and 14 different SCD patients in steady state (repeat samples from the same patient were taken at least 6 months apart) from UCLH (Figs. 1a–c, 2, 3 and 4), 5 steady state SCD patients from UHBWT (Fig. 1d,e) and 5 healthy volunteers (Figs. 2, 3 and 4). Full blood counts and hemoglobinopathy data on patients from UCLH, at the time research samples were taken, can be found in Supplementary Table 3.

### ImageStream analysis

Antibodies against the cytoplasmic and extracellular domains of GPA (BRIC163 and BRIC256, respectively)^18,19^, were labelled with either Alexa Fluor-488 or Alexa Fluor-647. Twenty microlitres of PFP was mixed with either an equal volume of 0.22 µm filtered flow-buffer [PBS 1% BSA, 0.05% sodium azide (all Sigma-Aldrich, UK)] containing fluorescent labelled antibodies or 80 µl of 0.22 µm filtered Annexin V binding buffer (Annexin-V-FLUOS Staining Kit, Sigma-Aldrich) containing fluorescent Annexin V. Samples were left for one hour on ice before analysis on an ImageStreamX Mark II imaging flow cytometer (Luminex, USA) using the INSPIRE acquisition software ISX (Luminex, USA) (https://www.luminexcorp.com/imagestreamx-mk-ii/#software). Samples were run with a low flow rate but high sensitivity, 60× magnification and a minimum of 100,000 events (up to 500,000) acquired. Speed beads were excluded from acquisition and all lasers were set to full power. Unstained samples were used as negative controls. Buffer only and buffer plus antibody controls were run to test background signal. Sizing was performed using a flow cytometry sub-micron reference kit (Thermo Fisher Scientific, UK). Data analysis was performed using IDEAS (version 6.2) software (Luminex, USA) (https://www.luminexcorp.com/imagestreamx-mk-ii/#software) and fluorescent gates were set using fluorescence minus one controls (see Supplementary methods).

### RCDP sorting

Samples were stained as described above for ImageStream analyses. BRIC256^+ve^/BRIC163^−ve^ RCDP were sorted using an Influx high speed fluorescence activated cell sorter with BD Sortware 1.2.0 (BD Biosciences, UK) (https://www.bdbiosciences.com/en-eu/instruments/research-instruments/research-cell-sorters/influx). Sort gates were based on fluorescence minus one controls.

### Spinning-disk confocal microscopy

Microscopy plates (384 MatriCal MGB101-1-2-LG-BiomatriCal) were coated in poly-l-lysine (Sigma-Aldrich) prior to addition of BRIC256^+ve^ RCDP, then examined using an Opera LX HCS spinning-disk confocal microscope (Perkin Elmer, UK) with a 60× (NA 1.2) water-immersion lens and analysed using Acapella software (Perkin Elmer, UK). Digital images were visualised using ImageJ (https://imagej.nih.gov/ij/docs/index.html). The size and shape of RCDP were determined using a custom MATLAB script developed by the Wolfson Bioimaging Facility, University of Bristol. The script uses a built-in circular Hough transform in XY axes to detect particles. The 3D shape was determined by fitting an active contour to the radial average of the image, centred on the central Z-axis of each particle. The Hough transform tool is selective for objects with a high degree of radial symmetry and will ignore ellipses.

### Isolation and removal of RCDP

RCDP were isolated or removed from PFP by magnetic bead separation (Miltenyi Biotec, UK) in accordance with the manufacturer’s instructions (for full method see supplemental methods). To remove right-side out RCDP 40 µl anti-GPA magnetic beads (Miltenyi Biotec) were added to 800 µl PFP and incubated with rotation at 4 °C for 30 min. Inside-out RCDP were isolated using antibodies against the cytoplasmic domains of anion exchanger-1 (AE-1) (BRIC132) and GPA (BRIC163). PFP and beads were passed through MS columns (Miltenyi Biotec), in a magnetic field, under gravity and collected. For clotting assays, RCDP retained on the column were eluted into PFP by flushing 800 µl of PFP from a healthy individual through the column outside the magnetic field. Large RDCP were removed by 1.2 µm filtration.

### Transmission electron microscopy and immunolabelling (TEM)

Three microlitres of enriched right-side or inside-out RCDP were placed on a carbon coated copper grid, left to dry, then fixed in 4% (v/v) paraformaldehyde (Sigma-Aldrich) with 0.05% (v/v) glutaraldehyde (Agar Scientific, UK) at room temperature for 30 min. RCDP were washed in PBS, quenched in 20 mM glycine (Thermo Fisher Scientific) for 10 min and incubated with 1% acetylated BSA (Aurion, The Netherlands) in PBS for 10 min to block non-specific binding. Grids were placed in BRIC200 (anti-AE-1) (for right side-out vesicles) or BRAC66 (AE-1, N-terminal cytoplasmic domain for inside-out vesicles), for 1 h at room temperature then washed in 0.1% BSA PBS. Aurion conventional gold reagents (particle size 10 nm) were added to the grids. Anti-mouse gold particles were used for right side-out RCDP and anti-rat gold particles were used for inside-out vesicles. Unbound gold was removed by washing. Grids were counterstained with a solution of 0.3% (w/v) uranyl acetate (Biolab, UK) in 1.8% methylcellulose (Sigma-Aldrich) for 10 min on ice then air-dried using the wire loop method. Grids were examined on a Tecnai12 120 kV BioTwin Spirit transmission electron microscope (FEI Company, The Netherlands) and visualised using a FEI CETA camera and TIA software (FEI Company, The Netherlands).

### Clotting assay

Clotting assay was adapted from^32^. Two hundred microlitres of PFP was placed in a clear tube at 37 °C and left for 5 min. The time taken for a visible clot to form was timed. Clotting was activated by the addition of 20 mM Ca^2+^ and 1 µg of Factor Xa (New England Biolabs, UK) and PFP was swirled gently until a clot had visibly formed. The clotting time given for each sample was the average of three assays. Clotting assays were performed on PFP before and after RCDP removal to determine their effects on coagulation.

### Statistics

The Kruskall–Wallis test followed by Dunn’s post test was used to analyse differences in RCDP content in plasma from healthy individuals and from SCD patients in steady state and crisis. Two-way ANOVA followed by Holms-Sidak multiple comparisons test was used to compare BRIC256^+^ RDCP and BRC256^+^/PS^+^ RDCP from healthy individuals and from SCD patients in steady state and crisis. Changes in clotting times were analysed using paired two-tailed T tests. Comparisons of the fold difference in clotting times were conducted using unpaired T tests.

## Supplementary Information

Supplementary Information.
