# Supplementary material for: Large red cell-derived membrane particles are major contributors to hypercoagulability in sickle cell disease
Source: Sci Rep. 2021 May 26;11:11035. doi: 10.1038/s41598-021-90477-z (PMC8155063; doi:10.1038/s41598-021-90477-z)
Supplement: Supplementary file 1 — Supplementary Information. [file 41598_2021_90477_MOESM1_ESM.pdf]

Supplementary Information For

Large red cell-derived membrane particles are major contributors to hypercoagulability in sickle cell disease.

Rachel A. Smith[1,2,3], Tosti J. Mankelow[1,2], Despoina Drizou[1,2,4], Thomas Bullock[1], Tom Latham[1], Sara Trompeter[5], Allison Blair[1,2,4] and David J. Anstee[1,2].

1-Bristol Institute for Transfusion Sciences, National Health Service Blood and Transplant, Filton, Bristol, UK.
2 -NIHR Blood and Transplant Research Unit, University of Bristol, UK
3- School of Biochemistry, University of Bristol, UK.
4- School of Cellular and Molecular Medicine, University of Bristol, UK.
5-Joint Red Cell Unit, University College London National Health Service Foundation Trust, Haematology Department, London, UK.

Corresponding author: Tosti Mankelow, Bristol Institute for Transfusion Sciences, NHS Blood and Transplant, Northway, Filton, Bristol, BS34 7QH, UK. tosti.mankelow@nhsbt.nhs.uk. Tel:44 (0)1179217547, Fax:44(0)1179125796

Supplemental Methods

Monoclonal Antibodies

All monoclonal antibodies used in this study came from the Protein Development and Production Unit (PDPU) at NHS Blood and Transplant. BRIC256[1] and R10[2] have an extracellular glycophorin A (GPA) epitope, BRIC163[3] has an intracellular GPA epitope. BRAC66[4] and BRIC132[5] have intracellular AE-1 epitopes and BRIC200 has an extracellular AE-1 epitope.

Supplemental Table 1 Details of monoclonal antibodies used

Antibody	Origin	Epitope
BRIC163	Mouse	Intracellular GPA
BRIC256	Mouse	Extracellular GPA
R10	Mouse	Extracellular GPA
BRIC132	Mouse	Intracellular AE-1
BRAC66	Rat	Intracellular AE-1
BRIC200	Mouse	Extracellular AE-1

ImageStream®X Mark II imaging flow cytometry data analysis using IDEAS 6.2

ImageStream data was analysed using IDEAS version 6.2 (Luminex) with fluorescent gates set using fluorescence minus one controls. Raw image files were opened and a scatter plot of scatter intensity vs scatter max pixel was created to exclude speed beads from analysis and define low scatter and high scatter populations (Supplemental Figure 1A). To clean up these populations, ensuring that no large aggregates were present in the “low scatter” population and no small debris was present in the “high scatter” population, histograms of brightfield area were set up to exclude large area events and small area events from the “low scatter” and “high scatter” populations respectively (Supplemental Figures 1B and 1C).

Scatter plots were then created using the “low scatter-low area” gate, representing **low scatter events**, and the “high scatter-high area” gate, representing **high scatter events**. Scatter plots of channel 02 vs channel 11 fluorescence intensity were set up (channel 02 for Alexa Fluor 488 and channel 11 for Alexa Fluor 647). Gates were placed on these scatter plots using fluorescence minus one controls. Gated populations were observed in the image gallery to visually confirm that the gates were placed optimally.

Statistics were used from the two final scatter plots to obtain the number of “high scatter” and “low scatter” events that were positive for the respective antibodies or Annexin V (conjugated to Alexa Fluor-488 or Alexa Fluor-647), as described in figure legends. To calculate the number of events per μl, the volume of the sample processed by the ImageStream was obtained from Tools> Sample information> Focus/Fluidics.

Isolation of RCDP

RCDP were isolated by magnetic bead separation. Right side-out RCDP were removed from SCD PFP using anti-GPA MicroBeads (Miltenyi Biotec, UK). Inside-out RCDP were removed using anti-Cy5/Alexa Fluor-647 MicroBeads (Miltenyi Biotec) in conjunction with antibodies against the cytoplasmic domains of anion exchanger-1 (AE-1) (BRIC132) and GPA (BRIC163) conjugated to Alexa Fluor-647. Antibodies were filtered through a 15nm filter (Whatman International Ltd, UK) to remove aggregates. Microbeads (40μl) and, if appropriate, 5μg BRIC132 or BRIC163 were added to 800μl of SCD PFP and incubated with rotation at 4°C for 30 minutes. PFP and beads were passed through MS columns (Miltenyi Biotec), in a magnetic field, under gravity and collected. For clotting assays, RCDP retained on the column were eluted by flushing 800μl of PFP from a healthy individual through the column outside the magnetic field. For electron microscopy, RCDP were removed using 300μl of PBS buffer containing 0.5% (v/v) human serum albumen (Irvine Scientific, Ireland) and 0.6% (v/v) Citrate-phosphate-dextrose (Sigma-Aldrich). Large RCDP were removed by syringe-driven filtration of PFP through a 1.2μm polyethersulfone acrodisc filter (PALL Life Sciences, UK). PFP samples were assayed before and after RCDP removal on the ImageStream using BRIC256 Alexa fluor-647 to determine removal rates.

References for Supplemental Methods

1. Reid ME, Lisowska E, Blanchard D. Coordinator's report: glycophorin/band 3 and associated antigens. *Transfus Clin Biol.* 1997;4(1):57-64.
2. Anstee DJ, Edwards PA. Monoclonal antibodies to human erythrocytes. *Eur J Immunol.* 1982;12(3):228-232.
3. Okubo Y, Daniels GL, Parsons SF, et al. A Japanese family with two sisters apparently homozygous for Mk. *Vox Sang.* 1988;54(2):107-111.
4. Beckmann R, Toye AM, Smythe JS, Anstee DJ, Tanner MJ. An N-terminal GFP tag does not alter the functional expression to the plasma membrane of red cell and kidney anion exchanger (AE1) in mammalian cells. *Mol Membr Biol.* 2002;19(3):187-200.
5. Wainwright SD, Tanner MJ, Martin GE, Yendle JE, Holmes C. Monoclonal antibodies to the membrane domain of the human erythrocyte anion transport protein. Localization of the C-terminus of the protein to the cytoplasmic side of the red cell membrane and distribution of the protein in some human tissues. *Biochem J.* 1989;258(1):211-220.

List of abbreviations in main text

AE-1	Anion Exchanger 1
AV	Autophagic vesicle
BSA	Bovine serum albumin
EV	Extracellular vesicles
GPA	Glycophorin A
MaV	Macrovesicle
PBS	Phosphate buffered saline
PS	Phosphatidylserine
PFP	Platelet free plasma
RCDP	Red cell-derived particles
SCD	Sickle cell disease
TEM	Transmission electron microscopy

Supplemental Figures

Supplemental Figure 1

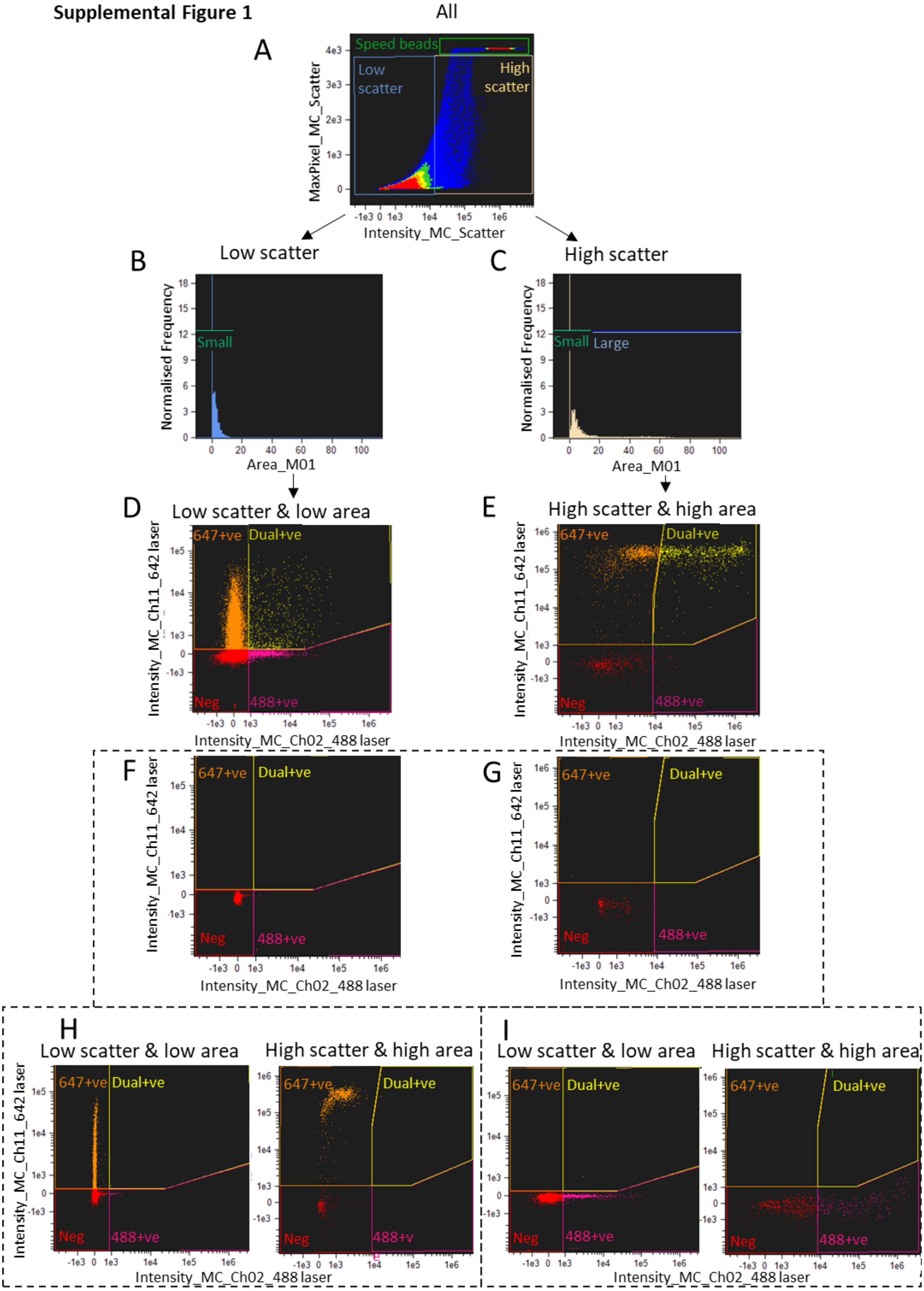

Supplemental Figure 1 ImageStream gating strategy

SCD plasma was processed and stained as described in the materials and methods section. (A) Scatter intensity (channel 06) was plotted against Scatter Max Pixel and gates were placed around speed beads (green), low scatter (blue) and high scatter (yellow) events. Low scatter events (B) and high scatter events (C) were plotted in a histogram of 'Brightfield Area' and a gate placed on low area events and high area events respectively. Scatter plots of channel 02 vs channel 11 fluorescence intensity were created for (D) low scatter & low area events (E) and high scatter & high area events. (D) and (E) show SCD PFP stained with both BRIC256 and BRIC163. For comparison also shown is (F) and (G) unstained, (H) BRIC256 stained and (I) BRIC163 stained from the same sample.

Supplemental Figure 2

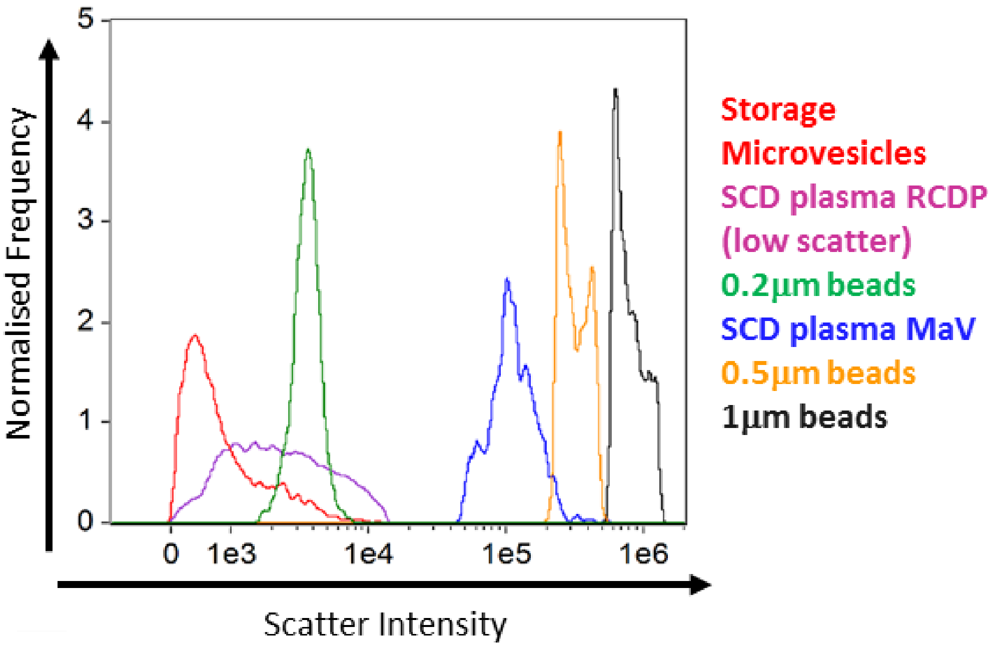

Supplemental Figure 2 Size determination of RCDP present in SCD plasma using size beads. SCD plasma was stained with BRIC256-Alexa Fluor 647 and BRIC163-Alexa Fluor 488 as described in the materials and methods section. MaV can be removed by filtration through a 1.2µm filter. SCD plasma was analysed on an ImageStream along with size beads. (A) Plot showing the scatter intensity of 0.2µm beads (green), 0.5µm beads (orange), 1µm beads (black), Erythrocyte storage induced EV (approximately 200nm) (red), SCD plasma RCDP (low scatter) (purple), and SCD plasma MaV (high scatter) (blue) on an ImageStream flow cytometer.

Supplemental Figure 3

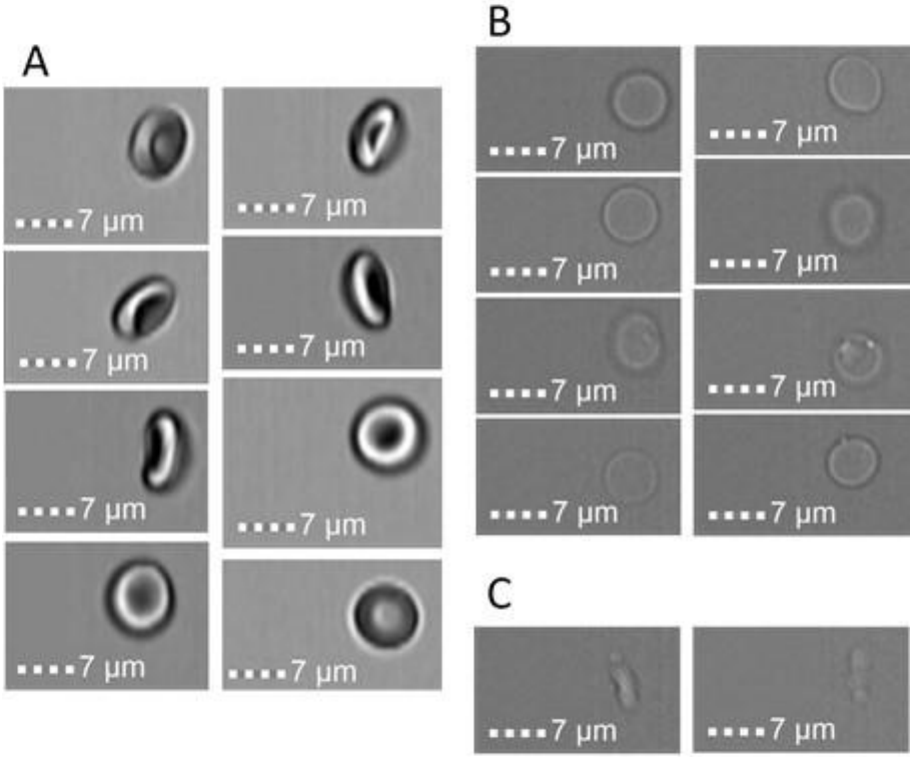

Supplemental Figure 3 Comparison of erythrocytes and MaV found in SCD PFP when viewed by Imaging flow cytometry.

When imaged with an ImageStream, erythrocytes are clearly different from the MaV observed in plasma from sickle cell patients. (A) Erythrocytes from a healthy individual look brighter and bolder compared with (B) MaV from SCD plasma. (C) Side views of MaV. Scale bars (7μm) are shown. Erythrocytes and MaV particles are of a similar size.

Supplemental Figure 4

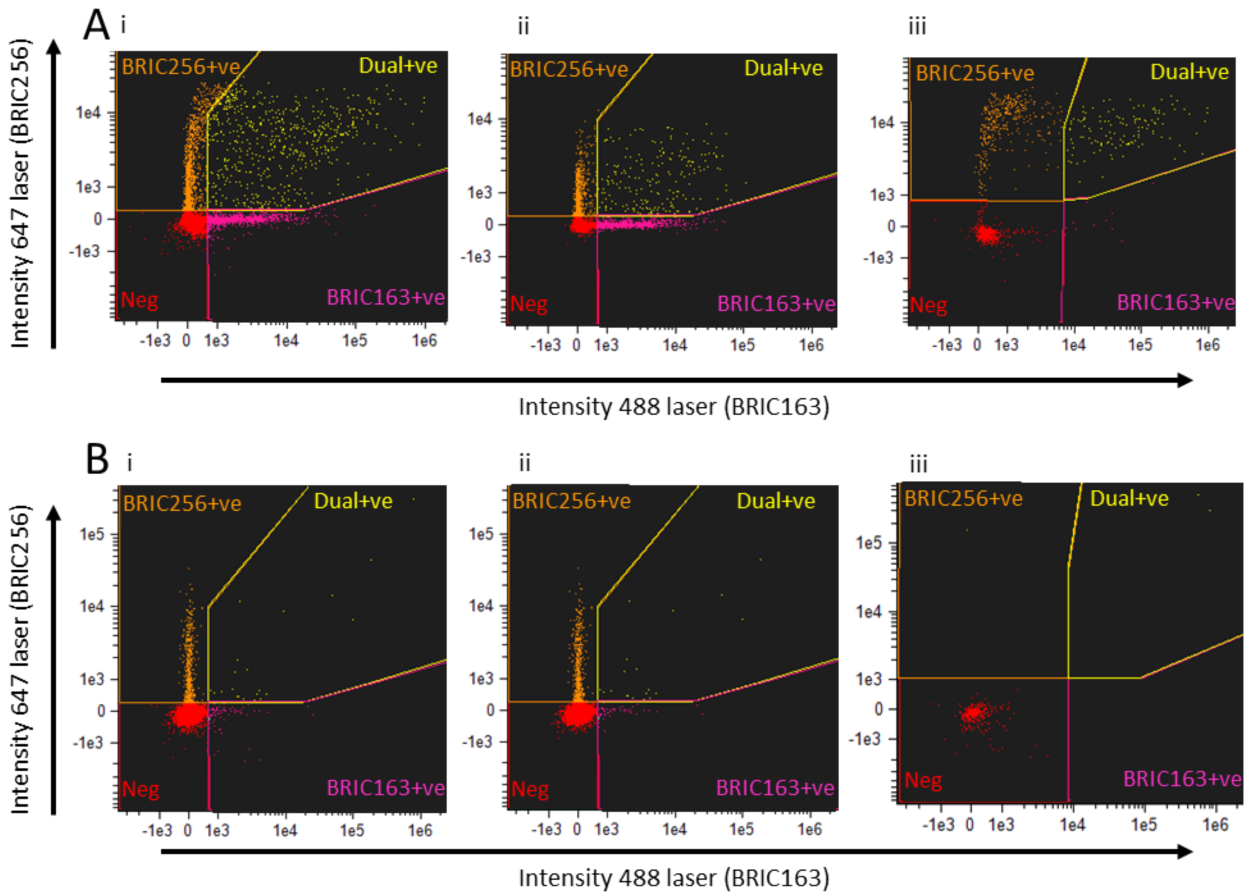

Supplemental Figure 4 Imaging flow cytometry analysis of SCD PFP and healthy PFP.

Plasma was processed and stained with BRIC256-Alexa Fluor 647 and BRIC163-Alexa Fluor 488, as described in the materials and methods. (A) The dot plots from Figure 1a showing SCD PFP stained with BRIC256 and BRIC163 (i) All RCDP detected, (ii) low scatter and (iii) high scatter. (B) For comparison identically stained healthy PFP (i) All RCDP detected, (ii) low scatter and (iii) high scatter.

Supplemental Figure 5

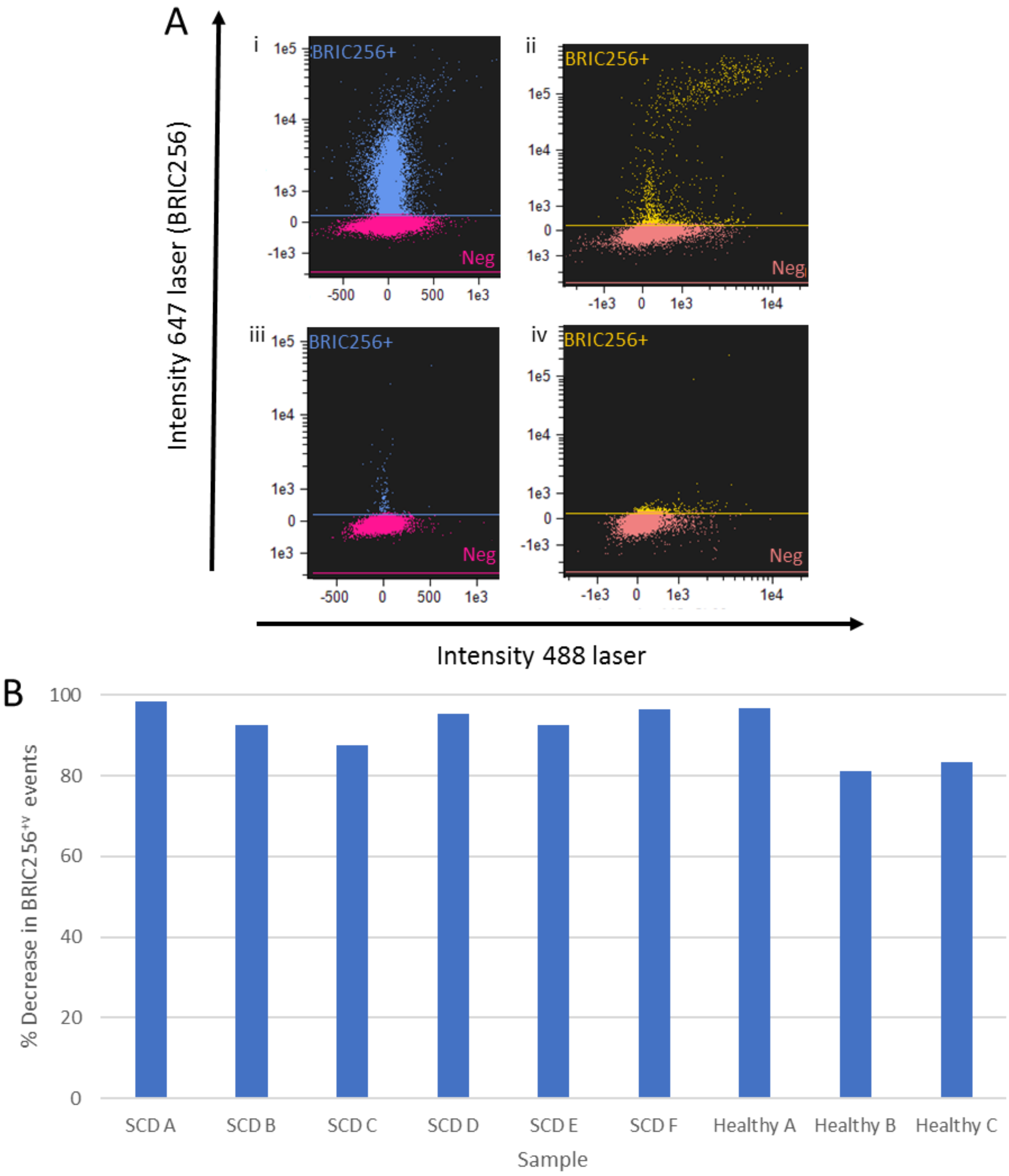

Supplemental Figure 5 Imaging flow cytometry analysis of SCD PFP before and after magnetic bead extraction

Right-side out RCDP were removed from PFP by anti-GPA magnetic bead extraction and the PFP stained with BRIC256-Alexa Fluor 647 before Imaging flow cytometry analysis (as described in materials and methods). (A) Analysis of low scatter particles detected (i and iii) and high scatter particles (ii and iv) pre extraction (i and ii) and post extraction (iii and iv). (C) Chart depicting percentage loss of events of total right-side out RCDP after anti-GPA magnetic bead extraction of six steady state SCD and three healthy PFP samples. This data demonstrates that anti-GPA magnetic bead extraction removes right-side out RCDP from SCD PFP.

Supplemental Figure 6

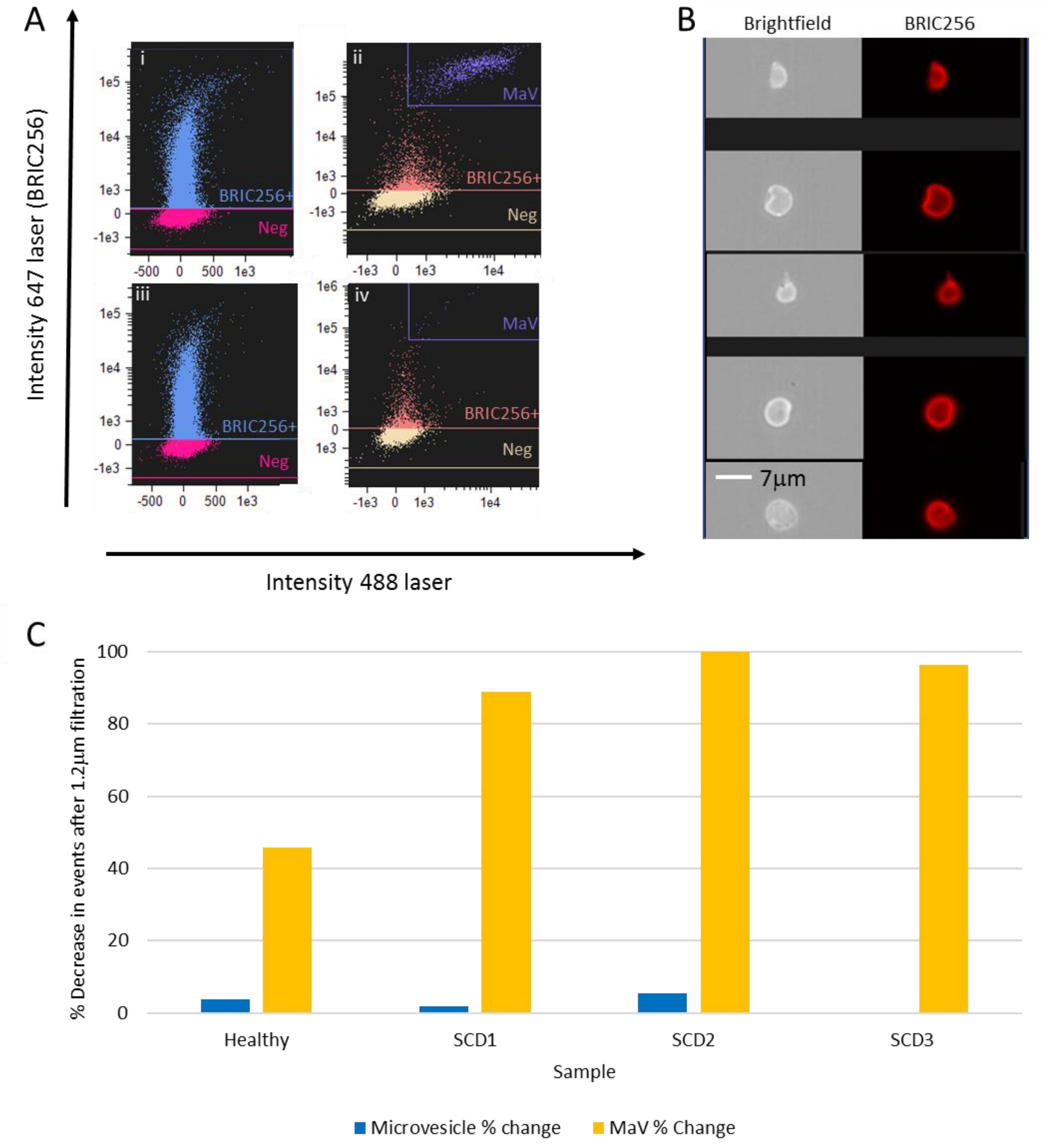

Supplemental Figure 6 Imaging flow cytometry analysis of SCD PFP before and after 1.2µm filtration.

SCD PFP was filtered through a 1.2µm syringe filter and stained with BRIC256-Alexa Fluor 647, as described in the materials and methods. (A) Analysis of low scatter particles detected (i and iii) and high scatter particles (ii and iv) pre filtration (i and ii) and post filtration (iii and iv). MaV particles are included in a separate gate in the high scatter population (MaV). (B) Brightfield and fluorescence images of the MaV population pre filtration. (C) Chart depicting

Supplemental Figure 6 legend continued.

percentage loss of events of RCDP (low scatter and non MaV high scatter) and MaV, detected by Imaging flow cytometry, after 1.2μm filtration of one healthy and 3 steady state SCD PFP samples. This data demonstrates that a 1.2μm filter removes the MaV population from SCD PFP.

Supplemental Table 2

	P1	P2	P3	P4	P5
Number of Values	1332	1547	150	113	183
Minimum	4.6	4.4	4.4	4.6	4.9
25% Percentile	6.4	6.2	6.8	6.7	6.5
Median	6.8	6.7	7.8	7.4	7.0
75% Percentile	7.2	7.2	8.5	8.6	7.5
Maximum	9.9	10.2	11.1	11.2	9.0

Supplemental Table 2 Spinning-disk confocal analysis of BRIC256 FACS sorted steady state SCD samples.

Scatter plot of size of each individual round object detected in each sample are shown in Figure 2C. Data range, median and quartiles are shown.

Supplemental Table 3 – UCLH patient information in figure order

Sample Number	Figure	Dot colour	Phenotype	Interval to previous transfusion		Type of previous transfusion		Sex	Free Hb (mg/l)*	Total bilirubin (μmol/l)
				Days	Weeks	Exchange	top up			
Crisis 1	2	Blue	HbSS	66	9.4	1		F	40.1	48
Crisis 2	2	Red	HbSS	165	23.6		1	F	375.4	43
Crisis 3	2	Yellow	HbSC					M	39.3	26
Crisis 4	2	Dark Purple	HbSS					M	44.3	31
Crisis 5	2	Dark Green	HbSS	1158	165.4		1	M	331.9	
Crisis 6	2	Brown	HbSS					M	64.4	73
Crisis 7	2	Light Green	HbSS					M	117.0	
Crisis 8	2	Cyan	HbSS	8	1.1		1	F	715.6	
Crisis 9	2	Light Purple	HbSS	153	21.9	1		M	46.8	
Crisis 10	2	Pink	HbSS	384	54.9	1		F	308.5	37
SS 1	2	Blue	HbSS	32	4.6	1		M	81.1	11
SS 2	2	Black	HbSS	31	4.4	1		M	6.7	8
SS 3	2	Red	HbSS	62	8.9	1		M	97.0	73
SS 4a	2	Yellow	HbSS	31	4.4	1		F		41
SS 5	2	Dark Purple	HbSD	32	4.6	1		M	168.9	58
SS 6a	2	Dark Green	HbSS	33	4.7	1		M	36.8	31
SS 7	2	Brown	HbSS	43	6.1	1		F	311.8	35
SS 8	2	Light Green	HbSS	35	5.0	1		M		37
SS 9	2	Cyan	HbSS	43	6.1	1		M		10
SS 10	2	Light Purple	HbSS	42	6.0	1		F	367.8	38
SS 11a	2	Pink	HbSS	42	6.0	1		M	81.1	24

Sample Number	Figure	Dot colour	Phenotype	Interval to previous transfusion		Type of previous transfusion		Sex	Free Hb (mg/l)*	Total bilirubin (μmol/l)
				Days	Weeks	Exchange	top up			
Crisis 1	3	Blue	HbSS	15	2.1			F	40.1	48
Crisis 2	3	Black	HbSS	243	34.7			F	375.4	43
Crisis 5	3	Red	HbSS	13	1.9			M	331.9	
Crisis 6	3	Yellow	HbSS					M	64.4	73
Crisis 7	3	Dark Purple	HbSS					M	117.0	
SS 1	3	Red	HbSS	32	4.6	1		M	81.1	11
SS 2	3	Blue	HbSS	31	4.4	1		M	6.7	8
SS 4a	3	Cyan	HbSS	31	4.4	1		F		41
SS 5	3	Light Purple	HbSD	32	4.6	1		M	168.9	58
SS 6a	3	Pink	HbSS	33	4.7	1		M	36.8	31
SS 7	3	Black	HbSS	43	6.1	1		F	311.8	35
SS 8	3	Dark Green	HbSS	35	5.0	1		M		37
SS 9	3	Light Yellow	HbSS	43	6.1	1		M		10
SS 10	3	Dark Purple	HbSS	42	6.0	1		F	367.8	38
SS 11a	3	Dark Yellow	HbSS	42	6.0	1		M	81.1	24

Sample Number	Figure	Dot colour	Phenotype	Interval to previous transfusion		Type of previous transfusion		Sex	Free Hb (mg/l)*	Total bilirubin (μmol/l)
				Days	Weeks	Exchange	top up			
SS 1	4a		HbSS	32	4.6	1		M	81.1	11
SS 2	4a		HbSS	31	4.4	1		M	6.7	8
SS 3	4a		HbSS	62	8.9	1		M	97.0	73
SS 4b	4a		HbSS	28	4.0	1		F		26
SS 11a	4a		HbSS	42	6.0	1		M	81.1	24
SS 12	4a		HbSS	39	5.6	1		F	66.9	75
SS 6b	4b		HbSS	35	5.0	1		M	377.0	36
SS 11b	4b		HbSS	42	6.0	1		M	49.3	28
SS 13	4b		HbSS	55	7.9	1		M	591.9	96
SS 14	4b		HbSS	42	6.0	1		F	25.1	41

* Free Hemoglobin was assayed in a research laboratory using the Harboe spectrophotometric method from Cookson *et al* Vox Sang. 2004 Nov;87(4):264-71.

Samples a and b were from the same patient and taken at least 6 months apart

Supplemental Table 3 (continued) – UCLH patient information in figure order

Sample Number	Hb (g/L)	HbS (%)	HbA (%)	HbF (%)	HbS plus C (%)	HbS plus D (%)	MCV (fL)	Red cell count (10^{12}/L)	Reticulocyte (%)	HCT	MCH (pg)	MCHC (g/L)	RCDW (%)
Crisis 1	87	55.4	35.5	5.8			86.6	2.99	9.1	0.259	29.1	336	19.1
Crisis 2		92.1		4.3			90.0	2.11		0.19	32.7	363	21.1
Crisis 3	122	94.8		1.6	94.8%		78.1	4.29	5.02	0.335	28.4	364	18.4
Crisis 4	95	88.8		7.9			66.3	3.98	7.42		23.9	360	23.6
Crisis 5	89	81.9		15.1			94.7	2.63		0.249	33.8	357	19.3
Crisis 6	112	89.4		7.5			94.8	3.43		0.325	32.7	345	18.4
Crisis 7	110	88.3		7.8			89.0	3.47	6.1		31.7	356	17.5
Crisis 8	100	21.5	75%	0.9			90.8	3.48	0.8		28.7	316	20.2
Crisis 9	98	85.1		11.3			82.8	3.31		0.274	29.6	358	21.2
Crisis 10	87	85.1		12.0			88.4	2.67	8.98	0	32.6	369	19.7
SS 1	105		60.4	3.4			88.9	3.50		0.311	30	338	3.5
SS 2	98	35.7	61.4				71.2	4.38		0.312	22.4	314	
SS 3	106	54.6	40.8	1.7			79.2	3.99		0.316	26.6	335	23.9
SS 4a	87	23.8	71.1	2.5			89.3	2.90	13.79	0.259	30	336	17.3
SS 5	115		48.5			48.7	80.8	4.28		0.346	26.9	332	19.2
SS 6a	95		51.1				64.2	4.55		0.292	20.9	325	
SS 7	81	32.5	62.5	2.1			85.3	2.72		0.232	29.8	349	19.6
SS 8	120	34.8	61.9	0.5			78.6	4.77		0.375	25.2	320	
SS 9	99	48.0	49.0				63.0	5.14		0.324	19.3	306	31.1
SS 10	87	37.9	55.9	3.5			83.2	2.98		0.248	29.2	351	17.8
SS 11a	105	41.8	53.4	1.2			81.0	4.00		0.324	26.3	324	19.8

Sample Number	Hb (g/L)	HbS (%)	HbA (%)	HbF (%)	HbS plus C (%)	HbS plus D (%)	MCV (fL)	Red cell count (10^{12}/L)	Reticulocyte (%)	HCT	MCH (pg)	MCHC (g/L)	RCDW (%)
Crisis 1	87	55.4		5.8			86.6	2.99	9.1	0.259	29.1	336	19.1
Crisis 2		92.1		4.3			90.0	2.11		0.19	32.7	363	21.1
Crisis 5	89	81.9		15.1			94.7	2.63		0.249	33.8	357	19.3
Crisis 6	112	89.4		7.5			94.8	3.43		0.325	32.7	345	18.4
Crisis 7	110	88.3		7.8			89.0	3.47	6.1		31.7	356	17.5
SS 1	105		60.4	3.4			88.9	3.50		0.311	30	338	3.5
SS 2	98	35.7	61.4				71.2	4.38		0.312	22.4	314	
SS 4a	87	23.8	71.1	2.5			89.3	2.90	13.79	0.259	30	336	17.3
SS 5	115		48.5			48.7	80.8	4.28		0.346	26.9	332	19.2
SS 6a	95		51.1				64.2	4.55		0.292	20.9	325	
SS 7	81	32.5	62.5	2.1			85.3	2.72		0.232	29.8	349	19.6
SS 8	120	34.8	61.9	0.5			78.6	4.77		0.375	25.2	320	
SS 9	99	48.0	49.0				63.0	5.14		0.324	19.3	306	31.1
SS 10	87	37.9	55.9	3.5			83.2	2.98		0.248	29.2	351	17.8
SS 11a	105	41.8	53.4	1.2			81.0	4.00		0.324	26.3	324	19.8

Sample Number	Hb (g/L)	HbS (%)	HbA (%)	HbF (%)	HbS plus C (%)	HbS plus D (%)	MCV (fL)	Red cell count (10^{12}/L)	Reticulocyte (%)	HCT	MCH (pg)	MCHC (g/L)	RCDW (%)
SS 1	105		60.4	3.4			88.9	3.50		0.311	30	338	3.5
SS 2	98	35.7	61.4				71.2	4.38		0.312	22.4	314	
SS 3	106	54.6	40.8	1.7			79.2	3.99		0.316	26.6	335	23.9
SS 4b	85	23.5	71.3	2.6			86.2	2.98		0.257	28.5	331	18.2
SS 11a	105	41.8	53.4	1.2			81.0	4.00		0.324	26.3	324	19.8
SS 12	75	27.1	70.1	0.2			88.8	2.60		0.231	28.8	325	20.5
SS 6b	97	45.7	51.5				67.2	4.64	1.92	0.312	20.9	311	
SS 11b	88	20.9	75.1	1.0			82.7	3.29		0.272	26.7	324	18.9
SS 13	83	24.5	72.4	0.3			86.8	2.96		0.257	28	323	19.1
SS 14	104	32.7	59.8	4.7			85.2	3.66		0.312	28.4	333	16.2

Abv - Hb (hemoglobin), HCT (Hematocrit), MCV (Mean cell volume), MCH (Mean Cell Hemoglobin), MCHC (Mean Corpuscular Hemoglobin Concentration) and RCDW (red cell distribution width).

Supplemental Table 3 (continued) – UCLH patient information in figure order

Sample Number	WCC (10^9/L)	Neutrophil (%WCC + 10^9/L)	Lymphocytes (%WCC + 10^9/L)	Monocytes (%WCC + 10^9/L)	Eosinophils (%WCC + 10^9/L)	Basophils (%WCC + 10^9/L)	Platelet count (10^9/L)	MPV (fL)
Crisis 1	12.76	73.8% 9.42	15.7% 2.00	9.4% 1.07	1.9% 0.24	0.2% 0.03	420	10.9
Crisis 2	10.14	59.0% 6.01	27.5% 2.79	8.9% 0.9	3.5% 0.35	0.8% 0.08	427	10.1
Crisis 3	16.44	80.0% 13.15	9.2% 1.51	10.6% 1.74	0.1% 0.02	0.1% 0.02	251	9.7
Crisis 4	13.78	64.2% 8.85	25.7% 3.54	8.7% 1.2	0.7% 0.1	0.7% 0.1	497	8.5
Crisis 5	23.30	34.9% 8.13	53.2% 12.40	10.1% 2.35	1.4% 0.33	0.4% 0.01	205	10
Crisis 6	12.93	68.0% 8.83	18.3% 2.37	11.6% 1.5	1.3% 0.17	0.5% 0.06	311	9.8
Crisis 7	9.42	48.8% 4.6	29.8% 2.81	20.3% 1.91	0.8% 0.08	0.3% 0.03	306	12.5
Crisis 8	11.50	68.1% 7.83	19.4% 2.23	8.9% 1.02	3.1% 0.36	0.5% 0.06	400	9.4
Crisis 9	16.30	70.2% 11.44	9.8% 1.60	18.3% 2.98	1.0% 0.16	0.7% 0.11	579	10.1
Crisis 10	8.06	38.9% 3.14	46.8% 3.77	11.2% 0.90	2.6% 0.21	0.5% 0.04	231	10.3
SS 1	6.80	57 .4 % 3.90	24.7% 1.68	14.0% 0.95	2.2% 0.15	1.8% 0.12	268	10.0
SS 2	10.19	60.1% 6.12	24.4% 2.49	14.0% 1.43	0.8% 0.08	0.7% 0.07	562	9.0
SS 3	11.84	63.9% 7.57	22.3% 2.64	10.4% 1.23	3.1% 0.37	0.35 0.04	359	10.4
SS 4a	8.88	74.1% 6.58	12.7% 1.13	11.5% 1.02	1.5% 0.13	0.2% 0.02	225	9.3
SS 5	9.10	56.0% 5.10	23.8% 2.17	16.6% 1.51	2.1% 0.19	1.5% 0.14	223	11.3
SS 6a	12.72	82.8% 10.53	9.7% 1.23	7.2% 0.92	0.08% 0.01	0.2% 0.03	237	
SS 7	10.64	52.2% 5.55	39.3% 4.18	5.9% 0.63	1.4% 0.15	1.2% 0.13	437	10.8
SS 8	9.82	56% 5.54	23.6% 2.32	11.8% 1.16	7.4% 0.73	0.8% 0.08	535	10.7
SS 9	6.67	62.5% 4.17	21.7% 1.45	12.6% 0.84	2.5% 0.17	0.7% 0.05	574	9.0
SS 10	8.93	43.1% 3.85	32.7% 2.92	14.4% 1.29	8.0% 0.71	1.8% 0.16	326	11.1
SS 11a	7.31	58.6% 4.28	20.8% 1.52	11.9% 0.87	7.7% 0.56	1.0% 0.07	346	12.0

Sample Number	WCC (10^9/L)	Neutrophil (%WCC + 10^9/L)	Lymphocytes (%WCC + 10^9/L)	Monocytes (%WCC + 10^9/L)	Eosinophils (%WCC + 10^9/L)	Basophils (%WCC + 10^9/L)	Platelet count (10^9/L)	MPV (fL)
Crisis 1	12.76	73.8% 9.42	15.7% 2.00	9.4% 1.07	1.9% 0.24	0.2% 0.03	420	10.9
Crisis 2	10.14	59.0% 6.01	27.5% 2.79	8.9% 0.90	3.5% 0.35	0.8% 0.08	427	10.1
Crisis 5	23.3	34.9% 8.13	53.2% 12.40	10.1% 2.35	1.4% 0.33	0.4% 0.01	205	10
Crisis 6	12.93	68.0% 8.83	18.3% 2.37	11.6% 1.50	1.3% 0.17	0.5% 0.06	311	9.8
Crisis 7	9.42	48.8% 4.6	29.8% 2.81	20.3% 1.91	0.8% 0.08	0.3% 0.03	306	12.5
SS 1	6.80	57 .4 % 3.90	24.7% 1.68	14.0% 0.95	2.2% 0.15	1.8% 0.12	268	10
SS 2	10.19	60.1% 6.12	24.4% 2.49	14.0% 1.43	0.8% 0.08	0.7% 0.07	562	9
SS 4a	8.88	74.1% 6.58	12.7% 1.13	11.5% 1.02	1.5% 0.13	0.2% 0.02	225	9.3
SS 5	9.10	56.0% 5.10	23.8% 2.17	16.6% 1.51	2.1% 0.19	1.5% 0.14	223	11.3
SS 6a	12.72	82.8% 10.53	9.7% 1.23	7.2% 0.92	0.08% 0.01	0.2% 0.03	237	
SS 7	10.64	52.2% 5.55	39.3% 4.18	5.9% 0.63	1.4% 0.15	1.2% 0.13	437	10.8
SS 8	9.82	56% 5.54	23.6% 2.32	11.8% 1.16	7.4% 0.73	0.8% 0.08	535	10.7
SS 9	6.67	62.5% 4.17	21.7% 1.45	12.6% 0.84	2.5% 0.17	0.7% 0.05	574	9.0
SS 10	8.93	43.1% 3.85	32.7% 2.92	14.4% 1.29	8.0% 0.71	1.8% 0.16	326	11.1
SS 11a	7.31	58.6% 4.28	20.8% 1.52	11.9% 0.87	7.7% 0.56	1.0% 0.07	346	12.0

Sample Number	WCC (10^9/L)	Neutrophil (%WCC + 10^9/L)	Lymphocytes (%WCC + 10^9/L)	Monocytes (%WCC + 10^9/L)	Eosinophils (%WCC + 10^9/L)	Basophils (%WCC + 10^9/L)	Platelet count (10^9/L)	MPV (fL)
SS 1	6.80	57.4 % 3.90	24.7% 1.68	14.0% 0.95	2.2% 0.15	1.8% 0.12	268	10.0
SS 2	10.19	60.1% 6.12	24.4% 2.49	14.0% 1.43	0.8% 0.08	0.7% 0.07	562	9.0
SS 3	11.84	63.9% 7.57	22.3% 2.64	10.4% 1.23	3.1% 0.37	0.35 0.04	359	10.4
SS 4b	8.81	75.2% 6.63	11.5% 1.01	10.0% 0.88	3.0% 0.26	0.3% 0.03	240	9.2
SS 11a	7.31	58.6% 4.28	20.8% 1.52	11.9% 0.87	7.7% 0.56	1.0% 0.07	346	12.0
SS 12	15.5	67.7% 10.49	16.5% 2.56	7.6% 1.18	7.3% 1.13	0.9% 0.14	417	11.0
SS 6b	14.9	75.1% 11.19	11.7% 1.74	10.7% 1.59	2.0% 0.30	0.5% 0.07	239	
SS 11b	8.25	66.0% 5.45	23.4% 1.93	7.9% 0.65	2.1% 0.17	0.6% 0.05	356	11.5
SS 13	11.41	51.4% 5.86	34.5% 3.94	10.9% 1.24	3.0% 0.34	0.2% 0.02	347	11.0
SS 14	11.56	81.5% 9.42	9.0% 1.04	8.3% 0.96	0.9% 0.10	0.3% 0.03	481	9.2

Abv – WCC (White Cell Count) and MPV (mean platelet volume).
